# No evidence for clonal transmission of urogenital carcinoma in California sea lions ( *Zalophus californianus*)

**DOI:** 10.12688/wellcomeopenres.11483.1

**Published:** 2017-06-22

**Authors:** Máire Ní Leathlobhair, Frances M.D. Gulland, Elizabeth P. Murchison

**Affiliations:** 1 Transmissible Cancer Group, Department of Veterinary Medicine, University of Cambridge, Cambridge, CB3 0ES, UK; 2 The Marine Mammal Center, 2000 Bunker Road, Sausalito, CA, 94965, USA

**Keywords:** California sea lion, urogenital carcinoma, transmissible cancer

## Abstract

Urogenital carcinoma is a highly metastatic cancer affecting California sea lions ( *Zalophus californianus*). The disease has high prevalence amongst stranded animals, and is one of the most commonly observed cancers in wildlife. The genital localisation of primary tumours suggests the possibility that coital transmission of an infectious agent could underlie this disease. Otarine herpesvirus type 1 has been associated with lesions, however a causative role for this virus has not been confirmed. We investigated the possibility that urogenital carcinoma might be clonally transmissible, spread by the direct transfer of cancer cells. Analysis of sequences at the mitochondrial DNA control region in seven matched tumour and host pairs confirmed that tumour genotypes were identical to those of their matched hosts and did not show similarity with tumours from other individuals. Thus our findings suggest that urogenital carcinoma in California sea lions is not clonally transmitted, but rather arises from transformed host cells.

## Introduction

Urogenital carcinoma (UGC) is the most commonly observed neoplasm in California sea lions ( *Zalophus californianus*) ^1^. This cancer was first reported in sea lions on the west coast of North America in 1979 ^2^, and over a fifteen-year period, from 1998 to 2012, the disease was found in 26 per cent of adult animals examined post-mortem at The Marine Mammal Center, California ^1^. UGC affects both male and female animals, and is most frequently found in sexually mature adults ^3, 4^. The disease typically presents with extensive multi-organ metastases; however, primary lesions involving the genital epithelium can usually be identified ^5^.

Three aetiological factors have been proposed for the development of UGC: infection, host genetics, and environmental factors. Otarine herpesvirus type 1 (OtHV-1), a gammaherpesvirus related to Kaposi’s sarcoma-linked human herpesvirus-8 ^5, 6^ has been associated with UGC ^5– 7^; however, this virus has not been confirmed as a causative agent. An association between UGC and genital bacterial infection has also been proposed ^8^. Genetic studies have indicated that individuals with high parental relatedness ^9^, homozygosity at the *HSPE2* locus ^10^, or one or more copies of the *Zaca-DRB.A* MHC class II locus ^11^ have increased risk of UGC. Environmental contaminants, such as organochlorines, have also been proposed as causative agents in UGC carcinogenesis ^12^.

Cancer occurs when a somatic cell acquires mutations that drive it towards a program of uncontrolled clonal expansion. Although cancer cells can migrate and invade distant tissues, most cancers remain within the body of the host that spawned them. Rarely, however, cancers can become transmissible such that cancer cells themselves become infectious agents that are transferred between individuals as allogeneic grafts. Only eight examples of naturally occurring contagious cancers are known: canine transmissible venereal tumour (CTVT) found in domestic dogs ^13, 14^, two distinct lineages of Tasmanian devil facial tumour disease ^15, 16^, and five lineages of disseminated neoplasia affecting various species of marine bivalves ^17, 18^. Tumours derived from clonally transmissible cancers carry the genetic material of the original animal that first gave rise to the cancer; thus, transmissible cancers are characterised by shared genotypes that are distinct from those of their matched hosts.

Several features of UGC are compatible with the possibility that this cancer is clonally transmissible: epidemiological observations of UGC are consistent with an infectious aetiology for the disease ^2^; and, in particular, its genital localisation could provide a coital route of transmission ^19^, as is observed with CTVT, the transmissible cancer in dogs.

We genotyped UGC tumours and their matched hosts to determine if UGC is clonally transmissible. Our results do not show evidence for UGC being a transmissible cancer, but rather confirm that UGC tumours are most likely derived from their hosts.

## Methods

### Ethics

This study was approved by The Marine Mammal Center Institutional Animal Care and Use Committee (Sausalito, CA) and the National Marine Fisheries Service MMPA (permit number 18786).

### Samples

Tissues from seven wild stranded adult California sea lions were collected at The Marine Mammal Center, Sausalito, CA. Complete gross and histopathological examinations were performed on each animal to confirm UGC diagnosis. Tumour (metastasis) and host tissue (liver or muscle) biopsies were collected into RNAlater during post-mortem examination and were stored at −70°C until processing.

### DNA extraction

Representative tissue sampled from tumour and host biopsies was used for DNA extraction using the Qiagen DNeasy Blood and Tissue Kit (Qiagen, Hilden, Germany) according to manufacturer’s instructions. DNA was quantified using a Qubit 2.0 fluorometer (Invitrogen, Carlsbad, CA, USA).

### PCR

We amplified a 1289 bp fragment of the mitochondrial DNA (mtDNA) control region using primers described by Wolf et al ^20^. PCR was performed using an Eppendorf Mastercycler Nexus GSX1 (Eppendorf, Hamburg, Germany) with conditions as follows: 40 ng of genomic DNA was amplified in a total volume of 20 μl containing 0.5 μM of each primer, 0.2 mM of each dNTP and 0.02 units of Taq DNA polymerase (Qiagen, Hilden, Germany) per reaction. Cycling conditions were 95°C for 3 min, 30 cycles of 95°C for 15 s, 60°C for 30 s, 72°C for 45 s and a final extension step at 72°C for 5 min. PCR products were purified using a QIAquick PCR purification kit (Qiagen, Hilden, Germany). Purified PCR products were capillary sequenced at Source BioScience LifeSciences Genomic Services (Source BioScience LifeSciences, Nottingham, United Kingdom).

### Alignment and variant calling

Sequences were aligned to the California sea lion mtDNA reference genome (accession number NC_008416) ^21^ using Sequencher DNA Sequence Analysis Software v5.4.6 (Gene Codes, Ann Arbor, MI, USA). Alignment errors were inspected manually and corrected. Variant positions were identified by viewing alignments, as well as by manual assessment of sequence chromatograms using FinchTV v1.4.0 (Geospiza Inc., Seattle, WA, USA). Variants were only assessed within a 397 bp region of the product, comprising region 15490–15886 in NC_008416.

## Results

We assessed 397 base pairs of the mtDNA control region in seven UGC tumours and their matched hosts. The analysis identified nine polymorphic sites characterising four unique genotypes within the sampled sea lion population ( Table 1). In all cases, the alleles present in tumours were identical to those found in matched host tissue ( Table 1). Chromatograms were closely examined at polymorphic sites, but no evidence for amplification of additional alleles in tumour tissues was found ^22^.

**Table 1.** Mitochondrial DNA (mtDNA) genotypes at nine polymorphic sites in sea lion hosts and matched tumours. Coordinates are relative to the sea lion mtDNA reference genome, NC_008416 ^21^. Individual California sea lions (CSLs) are labelled numerically and matched hosts and tumours are represented side-by-side. Alleles, represented by nucleotide code (A,C,G,T), are shown. Alleles that differ from the reference are shaded in grey.

	Individual:	Reference	CSL 1		CSL 2		CSL 3		CSL 4		CSL 5		CSL 6		CSL 7	
	Tissue:		Host	Tumour	Host	Tumour	Host	Tumour	Host	Tumour	Host	Tumour	Host	Tumour	Host	Tumour
**mtDNA coordinate**	**15524**	T	T	T	T	T	T	T	T	T	T	T	C	C	T	T
	**15527**	T	T	T	T	T	T	T	C	C	C	C	C	C	T	T
	**15528**	T	T	T	T	T	T	T	C	C	C	C	C	C	T	T
	**15550**	G	G	G	G	G	G	G	A	A	A	A	A	A	G	G
	**15551**	A	A	A	A	A	A	A	G	G	G	G	A	A	A	A
	**15629**	C	C	C	C	C	C	C	C	C	C	C	C	C	T	T
	**15652**	A	A	A	A	A	A	A	A	A	A	A	G	G	A	A
	**15660**	T	T	T	T	T	T	T	C	C	C	C	C	C	T	T
	**15812**	G	G	G	G	G	G	G	A	A	A	A	A	A	G	G

## Discussion

Our study does not support the hypothesis that UGC is clonally transmitted, but rather further confirms that UGC arises from host cells. Importantly, however, we cannot exclude the possibility that some UGCs are clonally transmitted. Given the genital localisation of this cancer, and likely accessibility of UGC cancer cells to other individuals during coitus, UGC tumours may pose a particular risk for the emergence of a transmissible cancer clone.

In this analysis we only examined genetic variation at one mtDNA locus. It is worth noting that at least one transmissible cancer – CTVT in dogs - has been observed to periodically capture mtDNA from its hosts ^23^; thus, mtDNA may not be considered the most reliable marker for testing clonality in transmissible cancers. However, mtDNA horizontal transfer events were detected only five times in a cohort of 449 CTVT tumours ^24^; thus even if mtDNA capture had occurred, it would not be expected that tumours would genetically match their hosts as frequently as we have observed in UGC.

Given that transmissible cancers are clonal lineages, tumour cell morphology and tissue architecture is generally very similar between tumours ^25, 26^. However, previous research has shown that UGCs appear to develop through histologically distinct stages ^5^, which further supports the idea of step-wise oncogenic transformation of host tissue rather than direct transmission of a cancer lineage.

Future research exploring the role of viral agents, host genetics and environmental factors, as well as somatic genetics, will be important for understanding the carcinogenic processes that cause UGC. It is interesting to note that an OtHV-1-associated UGC has recently been reported in a South American fur seal ( *Arctocephalus australis*) ^27^, indicating that other pinnipeds are susceptible to UGC, and further implicating OtHV-1 as a causative agent. Furthermore, a recent analysis of cytological smears collected from California sea lions in the Gulf of California revealed that transformation of the genital epithelium may be relatively common in this species ^28^.

Wildlife models of cancer can provide novel insights into general mechanisms of cancer development ^29^. Furthermore, an understanding of the aetiological factors underlying commonly observed cancers in wildlife is important for conservation and biomonitoring. In this study, we have found no evidence that UGC, one of the few “cancer epidemics” in wildlife ^1, 29^, is clonally transmitted. Ruling out this mode of carcinogenesis is an important step in our understanding of UGC, and paves the way towards further research investigating the processes underlying this aggressive disease in sea lions.

## Data availability

The data referenced by this article are under copyright with the following copyright statement: Copyright: © 2017 Ní Leathlobhair M et al.

Data associated with this study are available in GenBank with accession numbers: MF000998 - MF001011.
