# Supplementary material for: No evidence for clonal transmission of urogenital carcinoma in California sea lions ( Zalophus californianus)
Source: Wellcome Open Res. 2017 Jun 22;2:46. [Version 1] doi: 10.12688/wellcomeopenres.11483.1 (PMC5527528; doi:10.12688/wellcomeopenres.11483.1)
Supplement: Peer review history

# Peer review: Referee response for version 1

**DOI:** 10.21956/wellcomeopenres.12399.r23747

**Published:** 2017-07-17

**Authors:** Beata Ujvari

**Affiliations:** 1 Centre for Integrative Ecology, School of Life and Environmental Sciences, Deakin University, Waurn Ponds, VIC, Australia

Although many cancers are associated with infectious agents ^1^, only a few naturally occurring transmissible cancers have so far been identified in dogs, soft-shell clams and in Tasmanian devils. The lack of evidence for transmissible cancers can be related to several factors: (i) although neoplasia has been recorded in most metazoans, wildlife cancer statistics are, however, highly scattered in the scientific literature and hence challenging to access ^2^, (ii) lack of systematic screening (using large datasets) for the presence of clonally transmitted malignant cells in wildlife, (iii) failure to recognize extinct contagious cancers (that have been eliminated due to their detrimental impact on host fitness during the eons of evolution). Similar to pathogens/parasites, neoplasia (particularly communicable ones) can have a significant negative impact on host fitness in the wild and therefore be an important, but so far overlooked, evolutionary force that should not be ignored.  The study by Ní Leathlobhair *et al*. is one of the first, hopefully many more to come, systematic studies that attempts to identify the etiology of a wildlife cancer with high prevalence. The authors should be recommended for publishing negative results in search of wildlife transmissible cancers.

 In general, the paper would have benefited from higher sample sizes and the inclusion of more molecular markers into the analyses, although most likely the outcome of the paper, refuting the transmission hypothesis, would not have changed.

 One general concern with the article is basing the analysis solely on a fragment of mtDNA without acknowledging the potential hazard of amplifying nuclear mitochondrial DNA segments (NUMTs). NUMTs are insertions of mitochondrial DNA sequences into the nuclear genome, becoming pseudogenes, which are thought to be the remnants of gene transfer from mitochondria to nucleus ^3^. The transposition of mtDNA sequences into the nuclear genome has been documented in a wide variety of taxa, from fungi to insects to vertebrates ^4^. Although mounting evidence is suggesting that the occurrence of NUMTs is a ubiquitous phenomenon ^5^ studies frequently overlook their potential presence and generate unreliable results. NUMTs can be amplified in PCR reactions alongside their mitochondrial counterparts, becoming a source of contamination in mtDNA analysis ^6^. This is an issue that is often overlooked in studies of mtDNA and can result in misguided conclusions.  Although the outcome of the study by Ní Leathlobhair *et al.* would not have changed by the presence of NUMts, it would be so nice to see the scientific community finally acknowledging that simply amplifying fragments of mtDNA is not an acceptable scientific method to conduct evolutionary, population genetics, phylogenetic etc. studies.  There are several practices to screen for the presence of NUMTs, e.g. serial dilution method ^7^, nested approach to the PCR reactions ^8^, isolation of the mitochondrial genome during DNA extraction ^9^, digestion of nuclear DNA with DNase ^10^, sequencing of the entire mitochondria genome, for example through next generation sequencing (but see Verscheure, et al., 2015 ^11^, also Calvignac, S. et al. 2011 ^12^, Hazkani-Covo, E. et al. 2010 ^13^).

 In summary, the study by Ní Leathlobhair *et al*. is a nice contribution to the transmissible cancer literature, a delight to read, but I would highly encourage the authors of any future population genetic, phylogenetic and evolutionary studies to refrain from only analysing fragments of mtDNA, failing to amplify pure mtDNA can lead to unreliable results and erroneous conclusions. I would really like the practice of mtDNA fragment analysis (without eliminating NUMTs) to disappear from future scientific works.

I have read this submission. I believe that I have an appropriate level of expertise to confirm that it is of an acceptable scientific standard.

## References

[1] EwaldPWSwain EwaldHA: Infection and cancer in multicellular organisms. *Philos Trans R Soc Lond B Biol Sci*.2015;370(1673) : 10.1098/rstb.2014.0224 10.1098/rstb.2014.0224 26056368PMC4581029

[2] MadsenT. et al: Cancer Prevalence and Etiology in Wild and Captive Animals. In Ecology and Evolution of Cancer. *Academic Press*.2017;11-46

[3] WolffJNShearmanDCBrooksRCBallardJW: Selective enrichment and sequencing of whole mitochondrial genomes in the presence of nuclear encoded mitochondrial pseudogenes (numts). *PLoS One*.2012;7(5) : 10.1371/journal.pone.0037142 e37142 10.1371/journal.pone.0037142 22606342PMC3351421

[4] SorensonMQuinnT: Numts: A Challenge for Avian Systematics and Population Biology. *The Auk*.1998;115(1) : 10.2307/4089130 214-221 10.2307/4089130

[5] ZhangDXHewittGM: Nuclear integrations: challenges for mitochondrial DNA markers. *Trends Ecol Evol*.1996;11(6) :247-51 2123782710.1016/0169-5347(96)10031-8

[6] GoiosAPrietoLAmorimAPereiraL: Specificity of mtDNA-directed PCR-influence of NUclear MTDNA insertion (NUMT) contamination in routine samples and techniques. *Int J Legal Med*.2008;122(4) : 10.1007/s00414-007-0191-5 341-5 10.1007/s00414-007-0191-5 17874117

[7] DONNELLANS: Molecular evidence for the phylogeny of Australian gekkonoid lizards. *Biological Journal of the Linnean Society*.1999;67(1) : 10.1006/bijl.1998.0291 97-118 10.1006/bijl.1998.0291

[8] KumazawaYEndoH: Mitochondrial genome of the Komodo dragon: efficient sequencing method with reptile-oriented primers and novel gene rearrangements. *DNA Res*.2004;11(2) :115-25 1544954410.1093/dnares/11.2.115

[9] WrightJJLeeSZaikovaEWalshDAHallamSJ: DNA extraction from 0.22 microM Sterivex filters and cesium chloride density gradient centrifugation. *J Vis Exp*.2009; 10.3791/1352 10.3791/1352 19767717PMC3150049

[10] ZimmermanEGAkinsDRPlanzJVSchurrMJ: A rapid procedure for isolating mitochondrial DNA. *Gene Anal Tech*.5(5) :102-4 284796610.1016/0735-0651(88)90004-0

[11] VerscheureSBackeljauTDesmyterS: In silico discovery of a nearly complete mitochondrial genome Numt in the dog (Canis lupus familiaris) nuclear genome. *Genetica*.2015;143(4) : 10.1007/s10709-015-9844-3 453-8 10.1007/s10709-015-9844-3 25991039

[12] CalvignacSKonecnyLMalardFDouadyCJ: Preventing the pollution of mitochondrial datasets with nuclear mitochondrial paralogs (numts). *Mitochondrion*.2011;11(2) : 10.1016/j.mito.2010.10.004 246-54 10.1016/j.mito.2010.10.004 21047564

[13] Hazkani-CovoEZellerRMMartinW: Molecular poltergeists: mitochondrial DNA copies (numts) in sequenced nuclear genomes. *PLoS Genet*.2010;6(2) : 10.1371/journal.pgen.1000834 e1000834 10.1371/journal.pgen.1000834 20168995PMC2820518

# Peer review: Referee response for version 1

**DOI:** 10.21956/wellcomeopenres.12399.r23745

**Published:** 2017-07-06

**Authors:** Nicolas Bierne

**Affiliations:** 1 CNRS‐UM‐IRD (French National Center for Scientific Research-UM- Institute of Research for Development), Institute of Evolutionary Science of Montpellier (ISEM), Montpellier, France

Leathlobhair *et al.* conducted a straightforward test of the hypothesis that sea lions urogenital carcinoma (UGC) could be a transmissible cancer. They sequenced the mtDNA control region of seven tumors and their respective host tissue. They observed four different haplotypes in their sample and in each of the seven comparisons the two sequences were identical. This was the case even after a close inspection of the chromatograms to verify the presence of a possibly under-amplified sequence. Leathlobhair *et al.* study therefore does not support the hypothesis that sea lions UGC is a clonally transmitted cancer.

 Transmissible cancer is thought a rare exception. However, this conclusion suffers from a lack of a rigorous appreciation of the effort invested to test for genetic differences between tumor and host cells. In the wildlife especially, it is difficult to know how often the hypothesis of transmissible cancer is tested. For this reason the publication of negative results is essential and needs to be promoted. Although sea lions UGC was a pertinent suspect with an infectious aetiology suspected for years and a possible route of transmission via sexual relationship as in CTVT, the hypothesis of clonal transmission has not been tested/published before the timely study of Leathlobhair *et al.*

 The paper is clearly written and addresses all the relevant issues. I would suggest the authors to take the opportunity of their publication to promote a more systematic examination of the hypothesis of cancer transmission, possibly with an associated database. One question raised by Leathlobhair *et al. *study is about the genotyping effort required to definitively reject direct infection by cancer cells. Here the authors analyzed only 7 pairs with only one mtDNA marker with low nucleotide diversity. They rightly discussed the issue of mitochondrial captures, already reported in CTVT, which would be required to explain their data under the transmission hypothesis. However, only three captures is required to generate the four haplotypes observed in tumors. What makes the transmission hypothesis unlikely is the perfect match observed for rare haplotypes in CSL 6 and CSL 7. Nonetheless the authors could have provided a better idea of the nucleotide diversity observed in a wider sample of sea lions by using published data (eg in Gonzalez-Suarez *et al.* 2009 Mol Ecol, Schramm *et al.* 2009 Mar Biol). In addition, horizontal transfer has been found rare in the two mammalian transmissible cancers found to date, but this can be different for another new one. Riquet *et al* (2017) found 5 chimeric mussels thought to be infected by transmissible neoplasia with a combination of a nuclear SNP array and mtDNA sequences, but only two had the mtDNA of the transmissible lineage initially found in the pacific mussels by Metzger *et al.* (2016). The other three individuals had a perfect match with their host with mtDNA sequences while being chimeric at many nuclear SNPs. With only those three mussels and the use of mtDNA sequences, they would have missed the hypothesis of transmissible cancer in their sample. I would therefore suggest the authors to encourage the use of more markers, even if I am convinced that this first screen mostly refutes the transmission hypothesis. Genotyping a handful of nuclear markers is easy nowadays and I would not like subsequent studies to content to use only mtDNA sequences. Finally, one can suspect not so unlikely that standard oncogenic transformation of host tissue can sometimes infect a new host with few success of subsequent propagation -e.g. killing the new host rapidly because already at the metastatic stage. We would have a combination of standard and transmissible cancer lineages that would need a more extensive examination.

 To conclude, I very much enjoyed this paper, the approach is straightforward, I think a test of genetic chimerism in such a serious candidate as sea lions UGC was more than timely, I mostly concur that the hypothesis of clonal transmission can be rejected, and I thank the authors to promote by this submission a systematic search of transmissible cancer in the wildlife and the publication of “negative” results.

I have read this submission. I believe that I have an appropriate level of expertise to confirm that it is of an acceptable scientific standard.

# Peer review: Referee response for version 1

**DOI:** 10.21956/wellcomeopenres.12399.r23744

**Published:** 2017-07-03

**Authors:** Michael Metzger

**Affiliations:** 1 Department of Biochemistry and Molecular Biophysics, Columbia University, New York City, NY, USA

The Ní Leathlobhair *et al. *paper is an interesting article investigating the etiology of a cancer with high prevalence in California sea lions, specifically testing the hypothesis that it could be due to a transmissible cancer. Findings of transmissible cancer in multiple species has raised the possibility that other cancers could be due to transmission of cancer cells, specifically those that occur at high prevalence and those that have a plausible route of transmission, as are the case with the sea lion urogenital carcinomas.

 The finding of mtDNA SNPs between individuals, but no evidence of a neoplasia-associated allele strongly suggests that this disease is not a transmissible cancer. Since this result relies on the lack of detection of additional alleles on the chromatograms at polymorphic sites, it would be good to see at least a few examples of images from the chromatograms. However, given the analysis of paired host/tumor samples, this should have been obvious if present, unless the tumor cells only represent a small fraction of the tumor sample. Nuclear loci could have been checked to confirm this finding, but, as stated in the paper, in order to have transmissible cancer with no differences in the mtDNA it would require an unprecedented amount of mitochondrial transfer.

 Minor issue: It is unclear what primers were used for the PCR. The methods says that a 1289 bp fragment was amplified, but the reference cited appears to use two primer pairs to amplify mtDNA, generating 625 and 500 bp products. This reference (Wolf *et al.*) includes a table of other primers, but doesn't appear to list the primers for these PCR reactions, but the Features sections in the GenBank submissions reported in the paper does. Since neither of the primer pairs reported in the Wolf *et al. *study match the size reported, it is unclear if these are the primers used, however. It should be simple to add the sequences of the primers used to clarify the methods, as there was only one PCR done in this study.

 Overall, the results were negative, but it is important and valuable to other researchers to publish them, as it is a plausible hypothesis to explain the etiology of this common cancer.

I have read this submission. I believe that I have an appropriate level of expertise to confirm that it is of an acceptable scientific standard.
